# Pulmonary artery banding: a 20-year experience at a tertiary care center in a developing country

**DOI:** 10.3389/fcvm.2024.1368921

**Published:** 2024-04-29

**Authors:** Racha Ghoussaini, Rana Zareef, Adham Makarem, Nour Younis, Sally Al Hassan, Issam El Rassi, Munir Obeid, Fadi Bitar, Mariam Arabi

**Affiliations:** ^1^ Department of Internal Medicine, American University of Beirut Medical Center, Beirut, Lebanon; ^2^ Department of Pediatric and Adolescent Medicine, American University of Beirut Medical Center, Beirut, Lebanon; ^3^ Division of Cardiac Surgery, Massachusetts General Hospital and Harvard Medical School, Boston, MA, United States; ^4^ Department of Medicine, Harvard Medical School, Boston, MA, United States; ^5^ Department of Pediatric Cardiac Surgery, Al Jalila Children’s Specialty Hospital, Dubai, United Arab Emirates; ^6^ Surgery Department, American University of Beirut Medical Center, Beirut, Lebanon; ^7^ Division of Pediatric Cardiology, Department of Pediatric and Adolescent Medicine, American University of Beirut Medical Center, Beirut, Lebanon

**Keywords:** pulmonary artery banding, congenital heart disease, congenital heart surgery, palliative surgery, congenital heart defect

## Abstract

**Aim:**

Pulmonary artery banding serves as an important palliative procedure used for the management of several congenital heart lesions. This study aims to describe a 20-year experience of pulmonary artery banding at a tertiary care center in a developing country.

**Methods:**

This is a retrospective chart review of patients who underwent pulmonary artery banding over a 20-year period between January 2000 and July 2020 in a tertiary care center in a developing country. Data regarding demographics, indications, diagnosis, echocardiographic findings, postoperative complications, hospital stay, and outcomes were recorded.

**Results:**

A total of 143 patients underwent pulmonary artery banding between 2000 and 2020, with a decrease from approximately 15 surgeries per year in 2012 to 1–2 surgeries a year in 2020. At the time of banding, the median age of patients was approximately 90 days [interquartile range, IQR, 30–150 days]. Four patients (2.8%) died during the band placement. No significant association was observed between baseline characteristics or type of heart defect at presentation and postoperative morbidity and mortality.

**Conclusion:**

Pulmonary artery banding remains useful in a subset of congenital heart lesions and as a surgical palliation, with relatively low mortality, allowing postponement of total correction to a higher weight. This technique continues to be valuable in developing countries or for heart surgical programs with limited resources.

## 1 Introduction

Congenital heart disease (CHD) represents the most common congenital anomaly and is estimated to complicate 4–50 cases per 1,000 annual live births (1). It includes a wide spectrum of defects, severities, and complexities.

Pulmonary artery banding (PAB) is one of the oldest surgical techniques employed in treating patients with congenital heart lesions. It was first described by Muller and Dammann in 1951 to treat a 5-month old infant with ventricular septal defect (2). It was developed to prevent excessive pulmonary blood flow. The technique gained wide attention and is extensively employed in treating neonates and young infants with CHD who require staged correction. Indeed, in the 1980s, this technique recorded remarkable improvements in the survival of neonates with simple or complex lesions (3). However, this technique has been associated with significant mortality, reaching up to 25% in some cases (4–6). This often requires a second surgical intervention to remove the band, which holds its own risks of mortality and complications.

Pulmonary artery banding has been performed less frequently over time (4, 7). Nowadays, it represents around 2% of the total congenital heart surgeries. In the current era, primary correction of the congenital heart anomaly has replaced the staged approach (3). Nevertheless, PAB remains a viable temporary measure prior to the main corrective surgery (8). It can be used in training of the left ventricle in some cases of transposition of the great arteries or as part of a staged approach in patients with hypoplastic left heart syndrome (3).

The Children's Heart Center at the American University of Beirut Medical Center is a referral center that treats simple and complex congenital heart lesions. This study aims to investigate the 20-year experience of pulmonary artery banding at our institution, a tertiary care center in a developing country. It explores the procedure's indications, short- and long-term outcomes, including mortality, and complications encountered following band insertion.

## 2 Materials and methods

After securing Institutional Review Board (IRB) approval (BIO-2020-0325), we conducted a single-center, retrospective review of the medical charts of all patients who underwent pulmonary artery banding over a 20-year period at our institution between January 2000 and July 2020. A total of 143 patients who underwent pulmonary artery banding at the Children's Heart Center (CHC) of the American University of Beirut Medical Center (AUBMC) were identified. All data related to patients' demographics, echocardiographic findings, congenital anomaly characteristics, operative record, complications, mortality, progress notes, and hospital and intensive care unit (ICU) course were collected.

### 2.1 Statistical analysis

Descriptive statistics for patients' demographics, medical characteristics, and complications are presented for continuous variables as medians with interquartile ranges and for categorical variables as frequencies with percentages. The association between baseline characteristics, the type of congenital heart lesion and syndromes with postoperative morbidity and mortality was evaluated. Continuous variables were compared across groups using Wilcoxon Rank Sum test. For categorical variables, either the Chi squared test or Fisher's exact test was used; the latter was used in analyses with expected absolute number of less than 5.

In cases with missing data, complete case analysis was performed excluding patients with missing values. Statistical significance was considered at a *p* value of less than 0.05. All analyses were performed using SAS 9.4 (SAS Institute, Cary, NC, USA).

### 2.2 Banding technique

**Figure 1:**
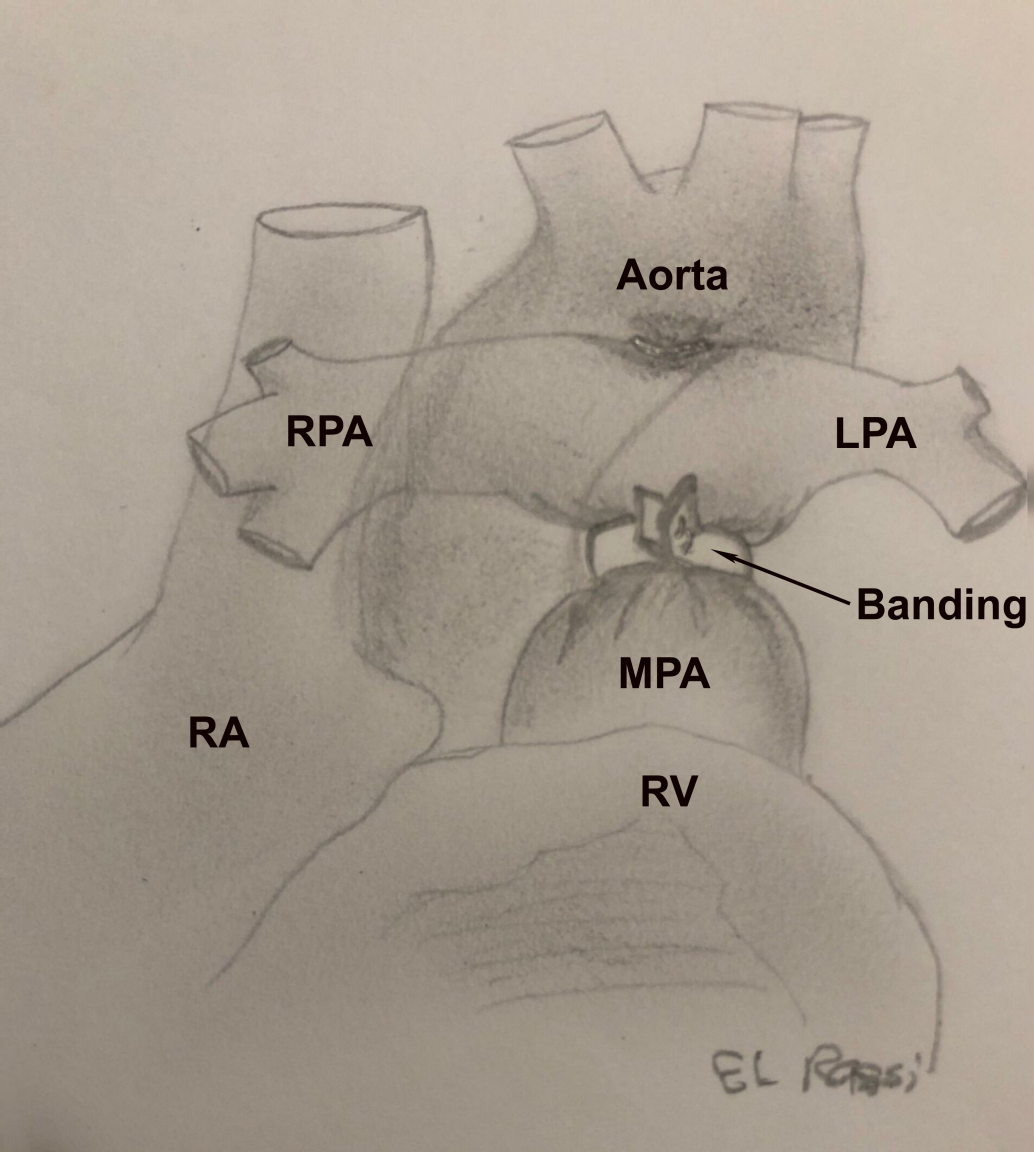
Illustration of the pulmonary artery banding.

The banding technique has been described in details in another study (9). Briefly, the procedure may be performed either through a thoracotomy or a partial or complete sternotomy. The pulmonary artery is then constricted with a band placed distal to the valve and proximal to the right pulmonary artery. The band is gradually tightened until the distal pressure drops by 30%–50% of the systemic pressure. Subsequently, the band is fixed to the adventitia layer to prevent migration. The pulmonary artery band is represented in Figure 1.

## 3 Results

**Figure 2:**
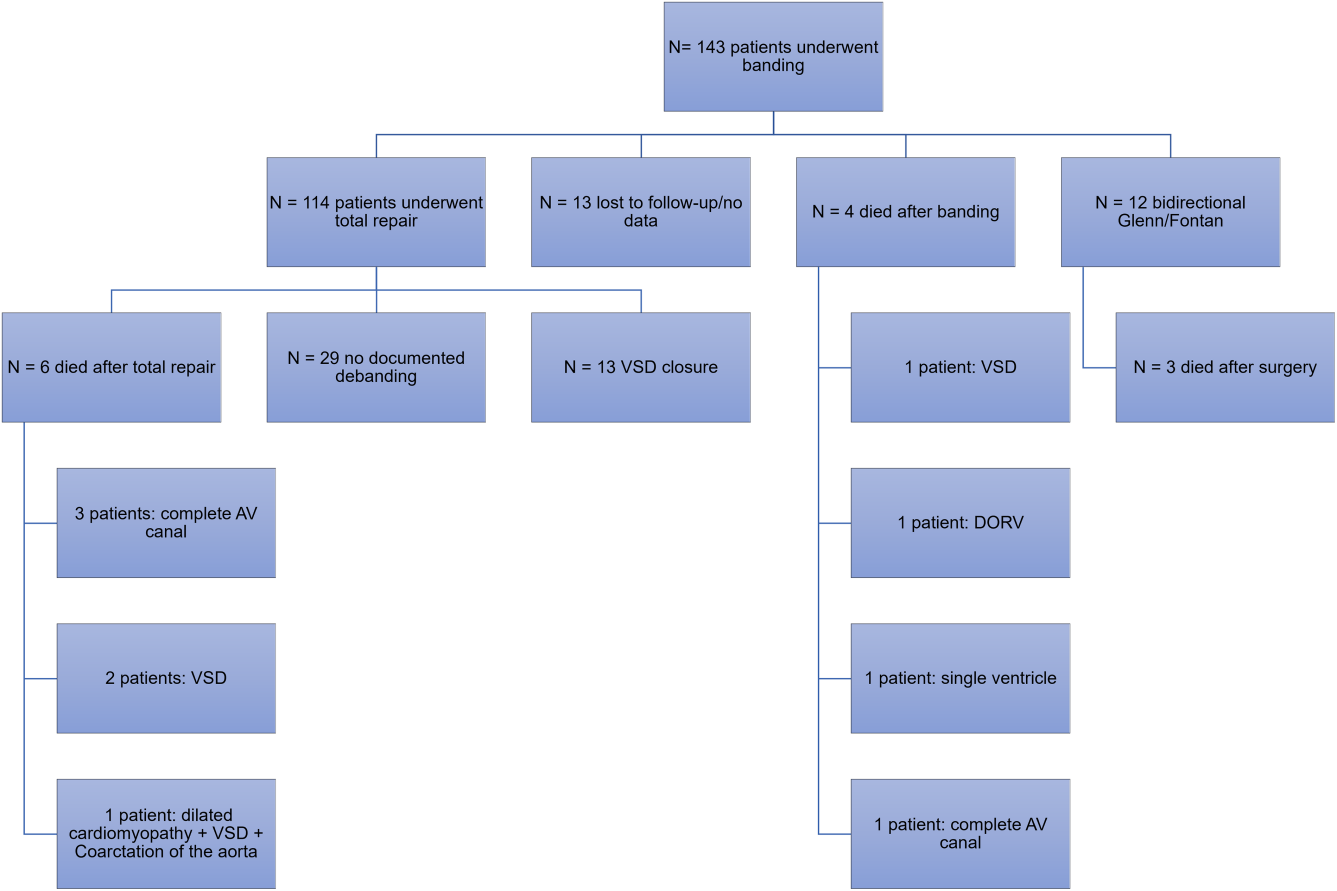
Flowchart of the study sample.

During the period of January 2000 and July 2020, a total of 143 patients underwent pulmonary artery banding at our institution. Among those, 114 patients (79.7%) underwent total repair with debanding and 12 patients (8.4%) underwent bidirectional Glenn and Fontan procedure. Among those who underwent total repair, 13 had ventricular septal defect (VSD) closure. Other surgeries included the hybrid stage II, surgical repair of the atrioventricular canal, pulmonary artery reconstruction and mitral valve repair, total anomalous pulmonary venous return repair, and coronary artery anomaly repair (Figure 2).

**Figure 3:**
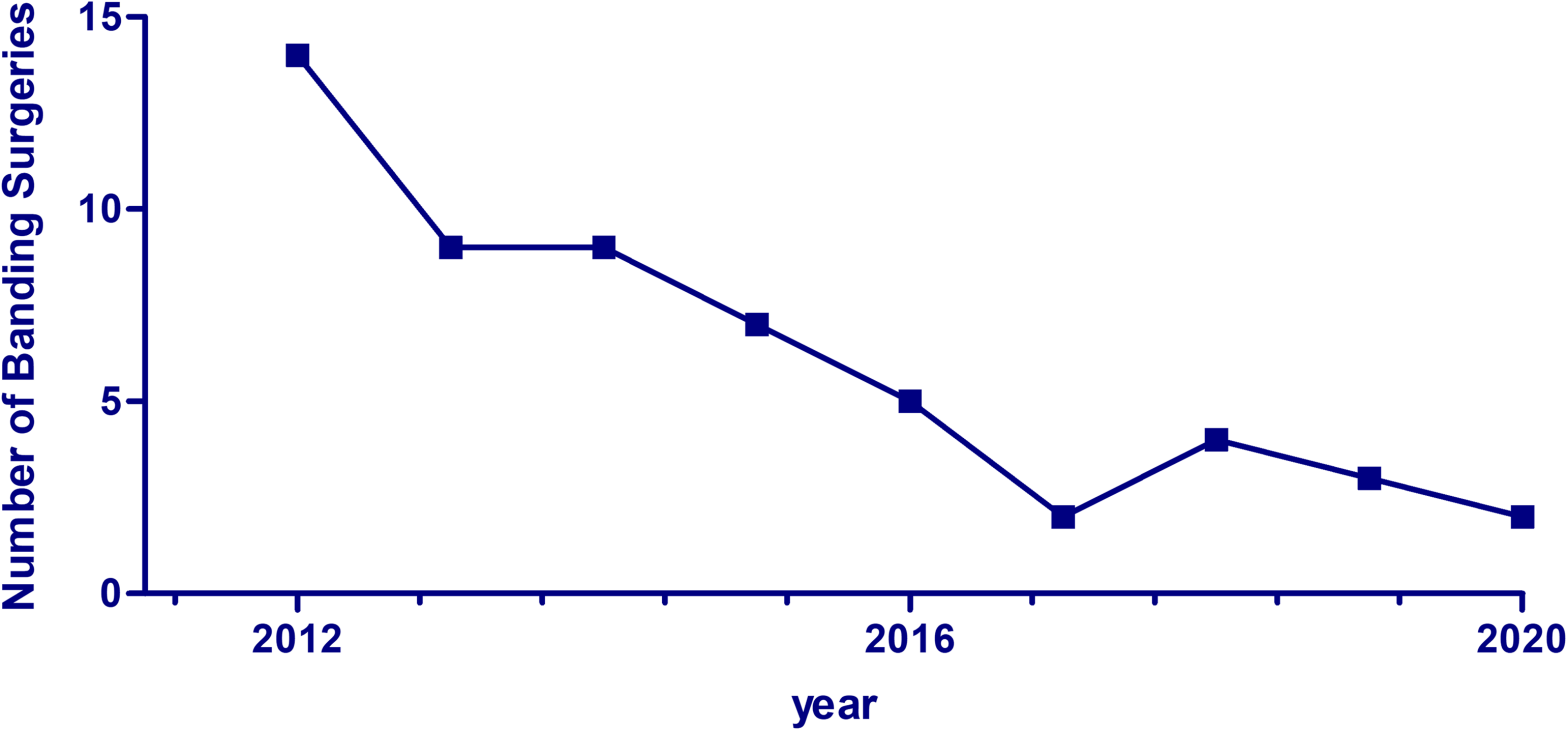
Annual number of pulmonary artery banding surgeries performed at our institution.

**Figure 4:**
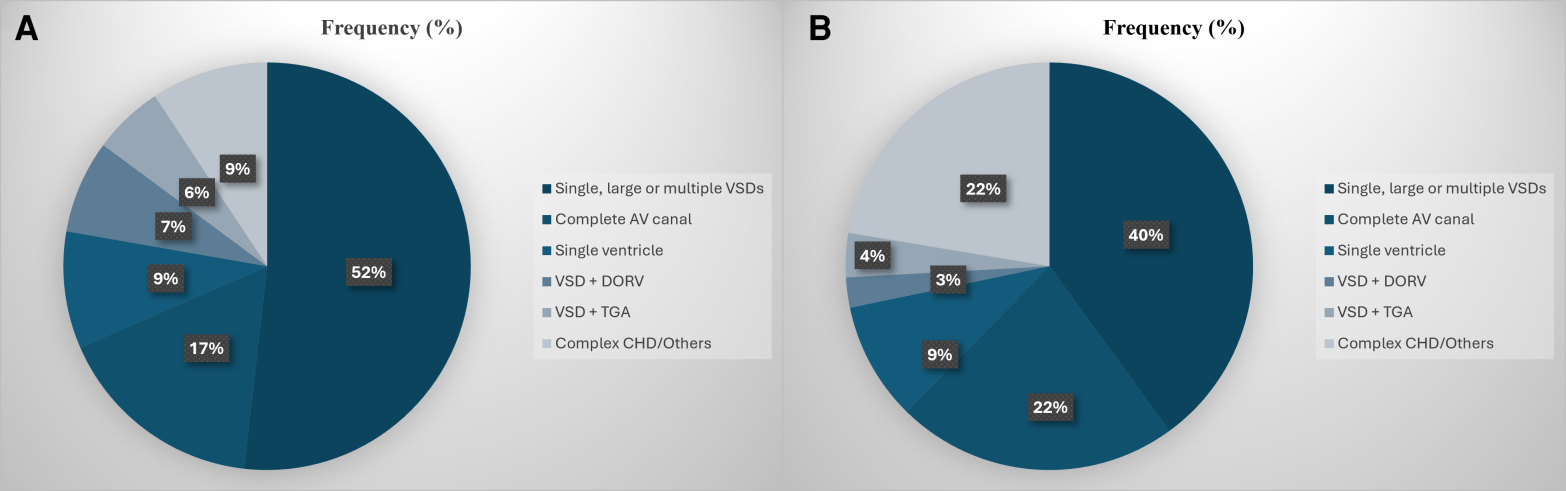
(**A**,**B**) Distribution of the pulmonary artery banding surgeries based on the structural heart lesion at diagnosis prior to 2010 (**A**) and from 2010 and onwards (**B**).

The percentage of pulmonary artery banding (PAB) surgeries out of the total number of surgeries performed annually at our institution declined from around 7.6% between 2000 and 2011 to 4.1% between 2012 and 2020, as shown in Figure 3. Prior to 2010, most of the banding surgeries (52%) were performed on patients with large or multiple VSDs. This was followed by complete AV canal (17%) and single ventricle (9%). From 2010 onwards, the proportion of PAB surgeries performed on patients with single or complex VSDs declined from 52% to 40%, while the frequency of surgeries performed on patients with a single ventricle remained stable at 9% (Figure 4).

As detailed in Table 1, the median age of patients at presentation was one day [IQR 0–30]. Seven (4.9%) patients were diagnosed prenatally, while 56 (39.8%) were diagnosed at birth. The median age of patients at the time of pulmonary artery banding was 90 days [IQR 30–150]. Of our sample, 44.6% were females, and 22.3% suffered from concomitant comorbidities. The most common comorbidities present at the time of pulmonary artery banding surgeries were syndromes (13.5%). This was followed by neurological diseases (3.4%), which include seizure disorder and neurodevelopmental delay, and other problems (3.4%), including but not limited to congenital hypothyroidism, G6PD deficiency, renal cysts, tracheoesophageal fistula, and others. Prematurity and congenital infections, including rubella and toxoplasmosis, were both the least frequent comorbidities (0.7%).

**Table 1:** Patients’ demographics, medical characteristics, and complications.

Demographics	*N* (%) or median [IQR]
Female	64 (44.6%)
Male to female ratio	1.2
Age at presentation (days)	3 [0, 30]
Age at banding (days)	90 [30, 150]
Comorbidities	*N* (%)
Syndromes	19 (13.5%)
Neurological diseases	5 (3.4%)
Other extra-cardiac issue	5 (3.4%)
Prematurity	1 (0.7%)
Congenital infections	1 (0.7%)
Characteristic	Median [IQR]
Age at total repair (months)	24 [12, 36]
Weight at total repair (kg)	10 [8, 12]
Time between banding and total repair (months)	18.5 [10, 25]
Post-operative data	Mean (±SD) or *N* (%)
Suspected or documented infection	14.2%
Length of hospital stay (days)	8 [7, 10]
Length of ICU stay (days)	3 [2, 6]
Days intubated	1 [0, 1]
Morbidity	18 (12.8%)
Death after banding	4 (2.8%)
Death after total repair/Glenn Fontan	5 (6.3%)

ICU, intensive care unit.

Following banding, patients either underwent total repair, during which debanding was performed, or bidirectional Glenn followed by the Fontan procedure for patients with a single ventricle. The median weight at total repair was 10 kg [IQR 8–12], and the median age was 24 months [IQR 12–36]. The median time between banding and total repair was 18.5 months [IQR 10–25]. Thirteen patients were lost to follow-up or had no documented information following placement of the pulmonary artery band. The above details are represented in Table 1.

Table 1 also describes the postoperative complications and outcomes observed following pulmonary artery debanding and total repair. 21 patients had a suspected or documented infection (14.2%). The median hospital stay was 8 days [IQR 7–10]. The median intensive care unit stay was 3 days [IQR 2–6]. 19 patients (12.8%) who underwent debanding and total repair experienced intraoperative and postoperative morbidity. Complications were noted but were rare and included postoperative ischemic strokes (*N* = 2), pneumothorax (*N* = 1), and hemothorax (*N* = 1). The in-hospital mortality during or after band placement was 2.8%. The mortality following total repair, debanding, and single ventricle surgeries was 6.3%.

There was no significant association between morbidity and mortality and sex, age at banding, and at total repair with debanding (Table 2). Similarly, the type of heart lesion had no significant correlation with morbidity and mortality (Table 2).

**Table 2:** Association between baseline characteristics, the most common structural heart lesions, syndromes and postoperative morbidity and mortality.

*N* = 77	Morbidity (at total repair)			Mortality (at total repair)		
	No 59 (76.6)	Yes 18 (23.4)	*p*-value	No 73 (94.8)	Yes 4 (5.2)	*p*-value
Sex, female; *n* (%)	25 (42.4)	8 (44.4)	0.88	31 (42.5)	2 (50)	0.77
Age at banding in months [Median (IQR)]	3.0 [1–5]	2.5 [1–4]	0.51	3.0 [1–5]	3.5 [2–5]	0.61
Age at total repair in months [Median (IQR)]	24 [12–36]	24 [12–24]	0.5	24 [12–36]	13 [12–19]	0.31
Heart lesions; *n* (%)			0.45			0.17
VSDs	28 (49.1)	11 (61.1)		38 (53.5)	1 (25)	
Complete AV canal	14 (24.6)	2 (11.1)		14 (19.7)	2 (50)	
Single ventricle	2 (3.5)	1 (5.6)		2 (2.8)	1 (25)	
VSD + DORV	5 (8.8)	0 (0)		5 (7.1)	0 (0)	
VSD + TGA	2 (3.5)	2 (11.1)		4 (5.6)	0 (0)	
complex CHD/others	6 (10.5)	2 (11.1)		8 (11.3)	0 (0)	

## 4 Discussion

This study aimed to describe the 20-year experience with pulmonary artery banding at the Children's Heart Center of American University of Beirut Medical Center, a tertiary care center in a developing country. First, and in accordance with the literature and the surgical experience worldwide, we have observed a significant decline over the years in the number of pulmonary artery banding surgeries, particularly between 2012 and 2016 (4, 10). This decline can be attributed to the replacement of the old, staged approach in managing complex congenital heart disease with early corrective surgery. This shift has been encouraged by the advances in surgical techniques, the refinement of cardiopulmonary bypass procedures (11), the presence of highly specialized cardiothoracic surgeons, and the improvements in the perioperative intensive care management.

Nonetheless, pulmonary artery banding continued to be performed until 2020 for various reasons, most commonly for palliation and pulmonary protection in cases of complex and multiple ventricular septal defects, in patients with single ventricle physiology, and for conditions where open-heart surgeries or total repair are contraindicated due to non-cardiac reasons or if they pose a high risk of mortality at an early age. Graziano and Agasthi revisited the current indications for banding in 2022, which include, but are not limited to: multiple or single VSDs with comorbidities, unbalanced atrioventricular septal defects with borderline left ventricular hypoplasia as a palliation before potential biventricular repair, as well as for left ventricular training as an adjunct in patients with single ventricle anatomy (12).

It is important to highlight that although total repair is now preferred over the staged approach, a staged approach starting with pulmonary artery banding as a surgical palliation in infants who cannot undergo open-heart surgery should still be considered. This strategy remains applicable in various regions, especially when dealing with high-risk neonates. For example, Nagashima et al. described their experience with using PAB in this population. They found that for infants weighing less than 2.5 kg, PAB had a lower mortality rate than single early total repair surgery and is therefore an effective temporary measure prior to total cardiac repair (13).

The estimated mortality after band placement at our institution was around 3%. This is consistent with previous studies (14). It's worth noting that the overall mortality rates following pulmonary artery banding surgeries have significantly decreased over the years (4, 14). For instance, in an investigation by Takayama et al. in 2002, the mortality for the group of patients who had a band placed between 1990 and 2001 was 13.8% (4), while the mortality during the second stage was around 6%. Furthermore, in our study, baseline characteristics at banding including age, sex and weight were not significantly associated with postoperative morbidity and mortality, consistent with previous literature (3).

Finally, 13 of our patients were lost to follow-up following pulmonary artery band placement. Although small, this fraction is significant when considering patients with congenital heart disease. This might be influenced by the financial constraints in our country, as is the case in many low- and middle-income countries, and the lack of proper national healthcare policy for patients with congenital conditions that would require lifelong follow-up. Extensive counseling regarding the importance and need for another surgery following band placement, as well as about the temporary nature of pulmonary artery banding, is crucial to minimize the loss to follow-up.

### 4.1 Limitations and strengths

This study is limited by its retrospective and observational design, as well as by the fact that data were collected from a single institution. Another potential limitation is the presence of missing data, which can be due to non-computerized charting during that period. Additionally, our study exhibits attrition bias, as some patients were lost to follow up. However, our study is consistent with the literature regarding indications for pulmonary artery banding and mortality rate, so it is tempting to assume that several findings may be also generalized to other settings.

## 5 Conclusion

Pulmonary artery banding has been utilized at our institution as part of a staged approach for correcting structural heart lesions and for palliation in certain cases. Our experience shows that banding remains a viable option when a staged approach is necessary in developing countries to manage infants at high risk of complications and mortality if subjected to cardiopulmonary bypass.

## Data availability statement

The original contributions presented in the study are included in the article/Supplementary Material, further inquiries can be directed to the corresponding author.

## Ethics statement

The studies involving humans were approved by Institutional Review Board at the American university of Beirut. The studies were conducted in accordance with the local legislation and institutional requirements. Written informed consent for participation was not required from the participants or the participants’ legal guardians/next of kin in accordance with the national legislation and institutional requirements.

## Author contributions

RG: Data curation, Formal Analysis, Writing – original draft. RZ: Data curation, Formal Analysis, Writing – original draft. AM: Writing – review & editing, Data curation, Formal Analysis, Validation. NY: Data curation, Writing – review & editing. SA: Data curation, Writing – original draft. IR: Conceptualization, Writing – review & editing, Methodology, Visualization. MO: Methodology, Data curation, Writing – original draft. FB: Conceptualization, Formal Analysis, Writing – review & editing. MA: Conceptualization, Validation, Writing – review & editing.
